# Automated Diffusion-Weighted MRI Analysis for Exploratory Risk Stratification of Malignant Cerebral Edema After Acute Ischemic Stroke

**DOI:** 10.3390/diagnostics16152483

**Published:** 2026-08-06

**Authors:** Cheng-Hsuan Juan, Chia-Hui Tsao, Ya-Hui Li, Chia-Ching Chang, Ming-Ting Tsai, Tung-Yang Lee, Cheng-En Juan, Hsiao-Wen Chung, Chun-Jung Juan

**Affiliations:** 1 Graduate Institute of Biomedical Electronics and Bioinformatics, National Taiwan University, Taipei 106, Taiwan; 2 Department of Medical Imaging, China Medical University Hospital, Taichung 404, Taiwan; 3 Department of Medical Imaging, China Medical University Hsinchu Hospital, Hsinchu 302, Taiwan; 4 Department of Management Science, National Yang Ming Chiao Tung University, Hsinchu 300, Taiwan; 5 Show Chwan Memorial Hospital, Changhua 500, Taiwan; 6 China Medical University Hospital, Taichung 404, Taiwan; 7 Master’s Program of Biomedical Informatics and Biomedical Engineering, Feng Chia University, Taichung 407, Taiwan; 8 Department of Electrical Engineering, National Taiwan University, Taipei 106, Taiwan; 9 Department of Radiology, School of Medicine, College of Medicine, China Medical University, Taichung 406, Taiwan; 10 Department of Biomedical Engineering and Environmental Sciences, National Tsing Hua University, Hsinchu 300, Taiwan; 11 Department of Computer Science and Information Engineering, National Taiwan University, Taipei 106, Taiwan

**Keywords:** stroke, ischemic, brain edema, diffusion magnetic resonance imaging, machine learning, risk stratification, image processing, computer-assisted

## Abstract

**Background/Objectives:** Malignant cerebral edema (MCE) is an infrequent but devastating complication of acute ischemic stroke (AIS). Early, reliable identification of patients at risk remains a major clinical challenge. The purpose of this study was to evaluate SCR-U1.8, a single automated imaging biomarker integrating stroke lesion burden and cerebrospinal fluid reserve, for exploratory risk stratification of MCE after AIS. **Methods:** In this retrospective study, consecutive patients with AIS who underwent diffusion-weighted imaging (DWI) between January 2019 and October 2022 were screened. Stroke lesions were automatically segmented on initial DWI using an ADC threshold of <1.8 × 10^−3^ mm^2^/s to derive U1.8 lesion volume. Cerebrospinal fluid volume (CSFV) was automatically estimated within the intracranial compartment, and SCR-U1.8 was calculated as U1.8/CSFV. Four low-complexity prediction strategies were evaluated: U1.8 > 82 mL, U1.8 > 145 mL, single-predictor U1.8 logistic regression, and single-predictor SCR-U1.8 logistic regression. Performance was evaluated across 100 repeated stratified patient-level train–test splits. Random undersampling was restricted to the training data, and no synthetic samples were generated. Model performance was assessed by accuracy, sensitivity, specificity, precision, negative predictive value (NPV), F1 score, receiver operating characteristic area under the curve (ROC-AUC), and average precision (AP) as appropriate. **Results:** A total of 530 patients were included, comprising 198 women and 332 men (mean age, 68 ± 14 years); a total of 12 patients developed MCE. Across 100 repeated stratified train–test splits, SCR-U1.8-LR achieved the highest accuracy (0.990 ± 0.006), precision (0.759 ± 0.150), specificity (0.992 ± 0.005), F1 score (0.806 ± 0.131), ROC-AUC (0.998 ± 0.002), and average precision (0.935 ± 0.062). Compared with the U1.8 > 82 mL threshold, SCR-U1.8-LR achieved significantly higher accuracy, precision, specificity, and F1 score, although sensitivity decreased from 1.000 to 0.900 and NPV decreased slightly from 1.000 to 0.998. **Conclusions:** SCR-U1.8 may provide a simple and physiologically interpretable measure of stroke lesion burden relative to CSF reserve. The findings remain exploratory because only 12 independent MCE events were available and require external validation.

## 1. Introduction

Malignant cerebral edema (MCE) is an uncommon but devastating complication of acute ischemic stroke (AIS), affecting approximately 2–5% of patients [1,2,3]. It is characterized by rapid neurological deterioration, elevated intracranial pressure, brain herniation, and high mortality [4,5,6]. Because timely interventions—particularly decompressive hemicraniectomy—can be life-saving when appropriately applied, early and reliable identification of patients at high risk is of substantial clinical importance [6,7,8].

Conventional predictive strategies primarily rely on fixed infarct volume thresholds derived from diffusion-weighted imaging (DWI). Seminal studies proposed thresholds such as greater than 145 mL within 14 h [9] or greater than 82 mL within 6 h after symptom onset [10]. However, these time-dependent, volume-based criteria are largely confined to the hyperacute phase, do not account for the dynamic evolution of edema, and fail to consider intracranial anatomy and compensatory reserve. As a result, generalizability and precision in broader clinical settings remain limited.

Recent advances in neuroimaging and machine learning provide opportunities for more refined risk stratification. DWI and apparent diffusion coefficient (ADC) maps enable the differentiation of cytotoxic and vasogenic edema components across a spectrum of ADC values. Automated lesion segmentation using deep learning techniques facilitates reproducible, observer-independent quantification of ischemic injury [11,12,13]. Automated quantification of U1.8 lesion volume provides a reproducible measure of total diffusion-abnormal lesion burden relevant to subsequent cerebral swelling. Beyond lesion volume alone, cerebrospinal fluid volume (CSFV) may reflect part of the intracranial compensatory reserve available to accommodate subsequent swelling. Importantly, in rare but high-stakes outcomes such as MCE, predictive precision is as critical as sensitivity. False-positive predictions may prompt unnecessary escalation of monitoring, repeat imaging, or clinical evaluation, thereby increasing patient burden and health care resource use.

We hypothesized that SCR-U1.8, which normalizes U1.8 lesion volume by CSFV, may provide more informative exploratory MCE risk stratification than lesion volume alone. This low-complexity approach was designed to evaluate lesion burden relative to available CSF reserve rather than lesion volume alone. Accordingly, the purpose of this study was to evaluate SCR-U1.8 as a single, automated imaging biomarker integrating stroke lesion burden and CSF reserve and to compare it with continuous U1.8 and established U1.8 volume thresholds.

## 2. Materials and Methods

This single-center, retrospective observational study was approved by the Research Ethics Committee, China Medical University & Hospital, Taichung, Taiwan (Approval No. CMUH114-REC2-129; approval date: 28 July 2025), with a waiver of written informed consent because all images and clinical data were fully anonymized. Definitions of imaging and clinical data elements with National Institute of Neurological Disorders and Stroke Common Data Elements are provided in Supplementary S1 (Table S1).

### 2.1. Patient Selection

**Figure 1:**
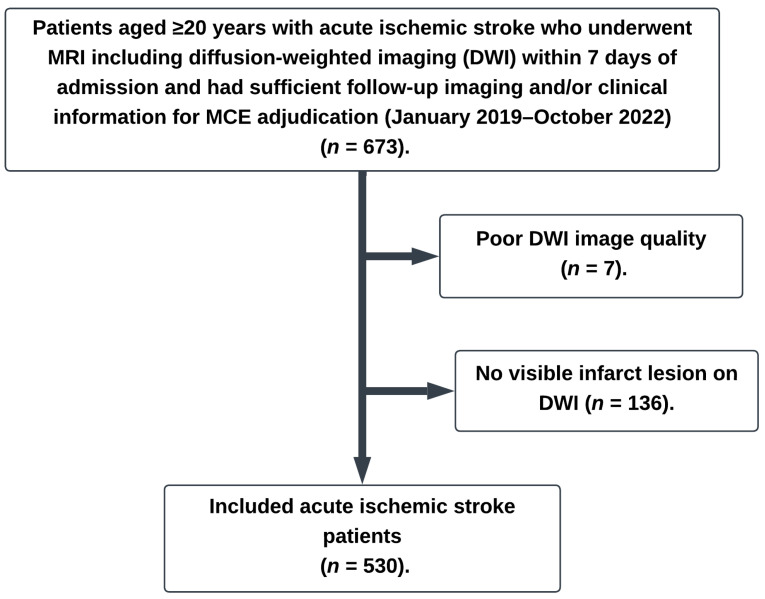
Flow diagram of patient selection. All included patients underwent baseline diffusion-weighted imaging within 7 days of admission and had sufficient follow-up imaging and/or clinical information for the adjudication of malignant cerebral edema. Follow-up CT or MRI was obtained when clinically indicated and was not required in every patient. MCE denotes malignant cerebral edema.

During the study period, MRI was not performed in all patients with AIS. Baseline MRI was obtained according to clinical judgment, institutional stroke workflow, patient stability, contraindications to MRI, and scanner availability. Follow-up CT or MRI was performed when clinically indicated, most commonly for neurological deterioration, the monitoring of large infarction, suspected edema progression, hemorrhagic transformation, or preoperative evaluation. All included patients had sufficient follow-up imaging and/or clinical information for adjudication of MCE; however, not all patients underwent a second neuroimaging examination. In some patients without MCE, the absence of MCE was established from subsequent clinical follow-up. Therefore, the analytic cohort represents a selected subgroup of AIS patients who underwent baseline DWI and had sufficient follow-up imaging and/or clinical information to determine MCE status. This potential selection bias was considered in the interpretation of the findings. Between January 2019 and October 2022, 673 consecutive patients aged 20 years or older with confirmed AIS who underwent DWI performed within 7 days of admission were screened by the first author (C.-H.J., with 4 years of experience in medical imaging research), who was blinded to the subsequent MCE outcome. Screening was limited to assessment of DWI image quality and confirmation of a visible acute infarction. Patients were excluded because of poor DWI image quality (*n* = 7) or absence of visible infarction on DWI (*n* = 136). The final analytic cohort comprised 530 patients, including 12 who developed MCE and 518 patients who did not (Figure 1).

### 2.2. Study Workflow

**Figure 2:**
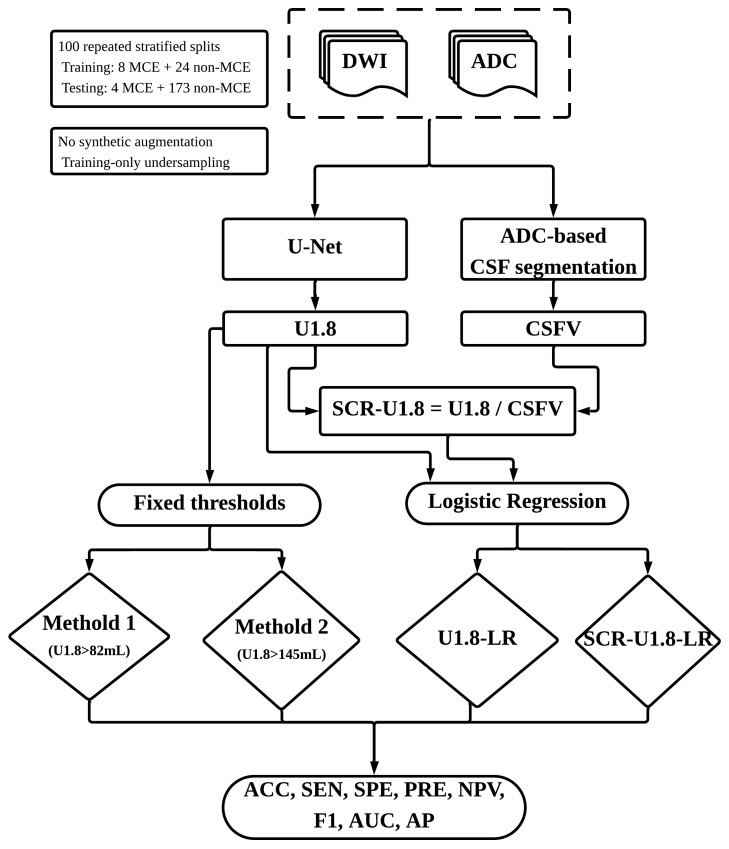
Overview of the automated DWI-based analysis and prediction workflow. Diffusion-weighted imaging (DWI) and apparent diffusion coefficient (ADC) maps were used for the automated quantification of U1.8 stroke lesion volume and cerebrospinal fluid volume (CSFV). SCR-U1.8 was calculated as U1.8/CSFV to represent stroke lesion burden relative to available CSF reserve. Four low-complexity prediction strategies were evaluated: predefined U1.8 thresholds of >82 mL and >145 mL and single-predictor logistic regression models using continuous U1.8 or SCR-U1.8. Performance was evaluated across 100 repeated stratified patient-level train–test splits. Random undersampling was applied only to the non-MCE training candidates, and no synthetic samples were generated. ACC, accuracy; SEN, sensitivity; SPE, specificity; PRE, precision; NPV, negative predictive value; F1, F1 score; AUC, area under the receiver operating characteristic curve; AP, average precision.

The study pipeline is shown in Figure 2.

Step 1: Image Acquisition

Clinical DWI and ADC maps were acquired according to the institutional stroke protocol.

Step 2: Ground Truth Determination

Two board-certified neuroradiologists, C.-J.J. and Y.-H.L., with 20 and 7 years of experience, respectively, independently adjudicated radiological MCE status using follow-up CT or MRI examinations and subsequent clinical information. MCE outcome adjudication was performed separately from the baseline DWI eligibility screening. The neuroradiologists were blinded to the automated U1.8 lesion volumes, CSFV measurements, SCR-U1.8 values, and model predictions. MCE was defined as midline shift >5 mm. This threshold was selected as an objective and reproducible indicator of clinically meaningful mass effect and substantial hemispheric swelling, while avoiding reliance on treatment decisions that may vary according to clinical judgment and institutional practice. Decompressive hemicraniectomy was recorded separately and was not included in the primary outcome definition. Disagreements were resolved by consensus. Inter-rater agreement was quantified using Cohen’s κ.

Step 3: Automated Stroke Segmentation

A previously validated U-Net model [11] was applied to DWI and ADC images to segment stroke lesions at an ADC threshold of <1.8 × 10^−3^ mm^2^/s. The resulting lesion volume was denoted U1.8.

Step 4: CSFV and SCR-U1.8 Calculation

CSFV was estimated within the intracranial compartment using morphological processing and an ADC threshold of >1.8 × 10^−3^ mm^2^/s [14,15,16,17,18]. SCR-U1.8 was calculated as U1.8/CSFV and represented stroke lesion burden relative to available CSF reserve.

Step 5: Repeated Resampling and Class Balancing

One hundred repeated stratified patient-level splits were generated. In each repetition, the training-candidate subset contained 8 MCE and 345 non-MCE patients, and the untouched testing subset contained 4 MCE and 173 non-MCE patients. Random undersampling was applied only to the training-candidate subset by retaining all 8 MCE patients and selecting 24 non-MCE patients, yielding a final training set of 32 patients. No synthetic samples were generated.

Step 6: Prediction Strategies

The predefined U1.8 thresholds of >82 mL and >145 mL were evaluated as historical benchmarks. Separate L2-regularized logistic regression models were fitted using continuous U1.8 or continuous SCR-U1.8 as the sole predictor. Continuous predictors were standardized using training-set parameters only.

Step 7: Cut-Point Selection

In each repetition, raw-feature cut points for U1.8 and SCR-U1.8 were selected from the training subset by maximizing the Youden index and were applied unchanged to the corresponding testing subset.

Step 8: Performance Evaluation

Model performance was evaluated in terms of classification performance and discrimination. Accuracy, sensitivity, specificity, precision, NPV, F1 score, ROC-AUC, and AP were calculated in the untouched testing set. Performance metrics were summarized across 100 repetitions using mean ± SD. Training-derived U1.8 and SCR-U1.8 cut points and Youden J values were summarized using medians and empirical 2.5th–97.5th percentile stability intervals.

Step 9: Statistical Analysis

Statistical procedures are described below.

### 2.3. Statistical Analysis

Statistical analyses were performed using Python 3.10.19. Continuous variables were compared between MCE and non-MCE groups using the Mann–Whitney U test, and categorical variables were compared using Fisher’s exact test. The comparison between SCR-U1.8-LR and U1.8-LR was prespecified as the primary comparison. Pairwise differences in performance metrics among all four prediction strategies were evaluated using the Wilcoxon signed-rank test across the 100 repeated stratified train–test splits. For each performance metric, all six pairwise comparisons were performed, and *p* values were adjusted using the Bonferroni method. Bonferroni-adjusted two-sided *p* values < 0.05 were considered statistically significant, corresponding to an unadjusted threshold of 0.0083. Because the repeated splits reused the same underlying patients and were not statistically independent, all comparisons and adjusted *p* values—including the prespecified primary comparison—were interpreted as exploratory.

## 3. Results

### 3.1. Patient Demographics

A total of 530 acute ischemic stroke patients were analyzed, including 198 women and 332 men, with a mean age of 68.33 ± 13.66 years (range: 26–100 years). Infarct territories included the anterior cerebral artery in 21 patients (4.0%), the middle cerebral artery in 373 (70.4%), the posterior cerebral artery in 66 (12.5%), and the vertebrobasilar artery in 115 (21.7%). Table 1 presents demographics of acute ischemic stroke patients specific for MCE and non-MCE groups. Interobserver agreement for MCE/non-MCE annotation was almost perfect (Cohen’s κ = 0.91), with discrepancies (2/12 MCE, 16.7%) resolved by consensus.

### 3.2. Feature Characteristics Between MCE and Non-MCE Groups

Table 2 compares the imaging measures central to the revised analysis. Patients with MCE had significantly larger U1.8 lesion volumes, lower CSFV, and higher SCR-U1.8 values than patients without MCE (all *p* < 0.001).

### 3.3. Training-Derived Cut Points

Table 3 summarizes the two prespecified literature-based U1.8 thresholds and the training-derived raw-feature cut points for U1.8 and SCR-U1.8. The fixed thresholds of U1.8 > 82 mL and U1.8 > 145 mL were retained as historical benchmarks and were therefore not associated with training-derived stability intervals. Across the 100 repeated stratified splits, the median training-derived U1.8 cut point was 106.89 mL, with an empirical 2.5th–97.5th percentile interval of 106.89–169.70 mL. The corresponding median SCR-U1.8 cut point was 1.43, with an empirical interval of 1.43–1.49. The relative interval width was 58.8% for U1.8 and 4.2% for SCR-U1.8, indicating less partition-to-partition variation in the SCR-U1.8 candidate cut point. The corresponding median training Youden J values were 0.957 for U1.8 and 0.994 for SCR-U1.8. However, because the repeated splits reused overlapping patients and each training subset contained only eight MCE cases, these intervals describe variability across repeated data partitions rather than confidence intervals derived from independent cohorts. The reported cut points should therefore be regarded as exploratory candidate thresholds rather than validated clinical decision thresholds.

### 3.4. Performance Comparison Across Repeated Train–Test Splits

Table 4 compares the performance of four prediction strategies across 100 repeated stratified train–test splits. The fixed U1.8 threshold of >82 mL achieved perfect sensitivity (1.000 ± 0.000) but relatively low precision (0.304 ± 0.058) and specificity (0.945 ± 0.013), indicating a comparatively high false-positive burden. Increasing the fixed threshold to >145 mL improved precision (0.448 ± 0.108) and specificity (0.975 ± 0.009) but reduced sensitivity to 0.810 ± 0.151.

The single-predictor U1.8-LR model showed intermediate performance, with sensitivity of 0.887 ± 0.196, precision of 0.339 ± 0.084, specificity of 0.958 ± 0.015, and an F1 score of 0.478 ± 0.095. In contrast, the SCR-U1.8-LR model achieved the highest accuracy (0.990 ± 0.006), precision (0.759 ± 0.150), specificity (0.992 ± 0.005), F1 score (0.806 ± 0.131), ROC-AUC (0.998 ± 0.002), and average precision (0.935 ± 0.062). Compared with the U1.8 > 82 mL threshold, SCR-U1.8-LR achieved significantly higher accuracy, precision, specificity, and F1 score, although sensitivity decreased from 1.000 to 0.900 and NPV decreased slightly from 1.000 to 0.998. Compared with the U1.8 > 145 mL threshold, SCR-U1.8-LR achieved significantly higher accuracy, precision, sensitivity, specificity, F1 score, and NPV. Compared with U1.8-LR, SCR-U1.8-LR achieved significantly higher accuracy, precision, specificity, F1 score, ROC-AUC, and average precision, whereas the differences in sensitivity and NPV were not statistically significant.

These results suggest that normalizing U1.8 by CSFV may improve discrimination and reduce false-positive classifications compared with U1.8 alone. However, because only 12 independent MCE events were available and the same cohort was reused across repeated splits, the findings should be interpreted as exploratory.

### 3.5. Case Demonstration

**Figure 3:**
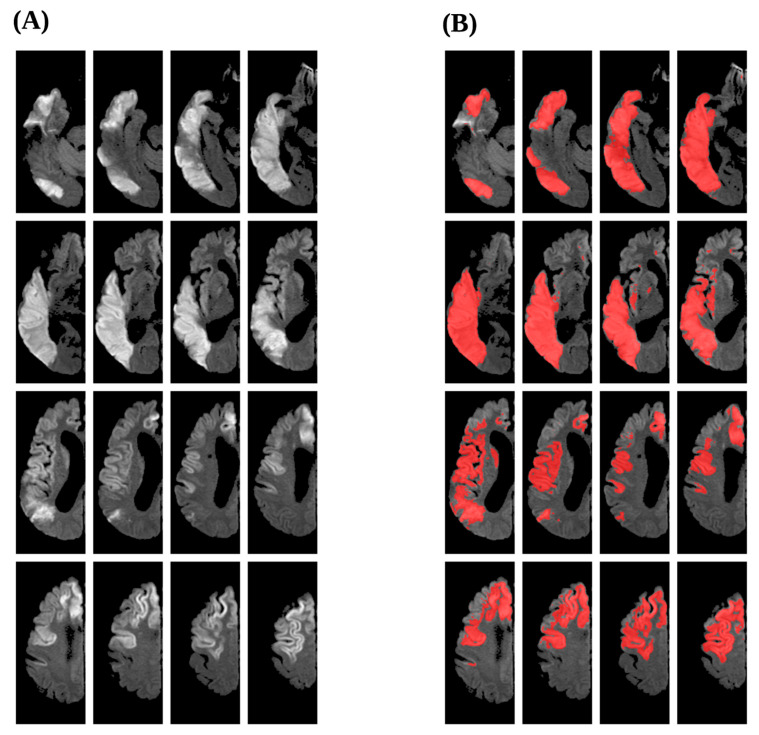
A 73-year-old man with an acute ischemic infarction involving the right frontal, temporal, and occipital lobes who did not subsequently develop malignant cerebral edema (MCE). (**A**) Diffusion-weighted imaging. The white area indicates the acute ischemic stroke lesion. (**B**) Overlay of the lesion segmentation generated by the U-Net U1.8 model, defined using an ADC threshold of <1.8 × 10^−3^ mm^2^/s, is shown in red. The patient had a U1.8 lesion volume of 173.87 mL, a relatively preserved CSFV of 163.52 mL, and an SCR-U1.8 of 1.063. The U1.8 > 82 mL threshold, U1.8 > 145 mL threshold, and U1.8-LR model incorrectly classified the patient as MCE, whereas the SCR-U1.8-LR model correctly classified the patient as non-MCE. This case illustrates how normalization of lesion burden by CSF reserve may reduce false-positive classification in patients with large infarcts.

Figure 3 presents a patient without MCE who had a U1.8 lesion volume of 173.87 mL, a CSFV of 163.52 mL, and an SCR-U1.8 of 1.063. The U1.8 > 82 mL threshold, U1.8 > 145 mL threshold, and U1.8-LR model all incorrectly classified the patient as MCE, resulting in false-positive classifications. In contrast, the SCR-U1.8-LR model, which incorporated lesion burden relative to the patient’s available CSF reserve, correctly classified the patient as non-MCE. This case illustrates that reliance on lesion volume alone may overlook interindividual differences in intracranial compensatory reserve and overestimate MCE risk in patients with large infarcts but relatively preserved CSF reserve.

**Figure 4:**
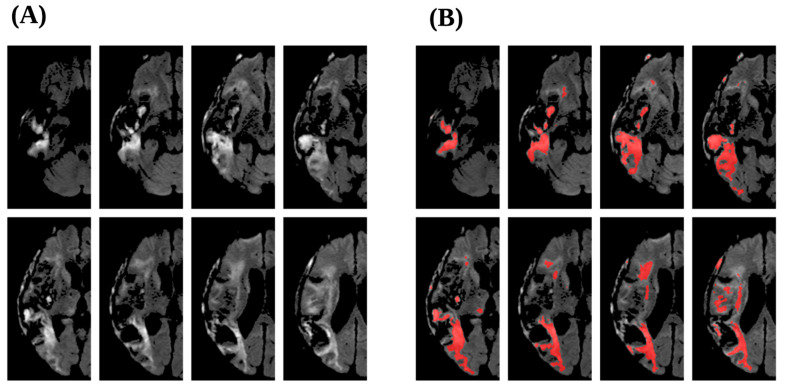
A 64-year-old woman with an acute ischemic infarction who subsequently developed malignant cerebral edema (MCE). (**A**) Diffusion-weighted imaging. The white area indicates the acute ischemic stroke lesion, whereas the black area indicates the area of hemorrhagic transformation. (**B**) Overlay of lesion segmentation generated by the U-Net U1.8 model, defined using an ADC threshold of <1.8 × 10^−3^ mm^2^/s, is shown in red. The patient had a U1.8 lesion volume of 106.89 mL, a CSFV of 71.64 mL, and an SCR-U1.8 of 1.492. The patient also had hemorrhagic transformation, and the hemorrhagic component was not included in the U-Net-based lesion segmentation; therefore, the measured U1.8 likely underestimated the total infarct burden. Although the U1.8 lesion volume did not exceed the conventional 145 mL threshold, the SCR-U1.8 value of 1.492 exceeded the median training-derived candidate cut point of 1.43. The U1.8 > 145 mL method classified the patient as non-MCE, whereas the SCR-U1.8-LR model correctly classified the patient as MCE. This case illustrates how incorporating CSF reserve may help identify high-risk patients whose measured lesion volume remains below a conventional large-volume threshold but is disproportionately high relative to their available intracranial compensatory reserve.

Figure 4 presents a patient with MCE who had a U1.8 lesion volume of 106.89 mL, a CSFV of 71.64 mL, and an SCR-U1.8 of 1.492. The patient also had hemorrhagic transformation, and the hemorrhagic component was not captured by the U-Net-based lesion segmentation, likely leading to the underestimation of the total infarct burden. The U1.8 > 145 mL threshold incorrectly classified the patient as non-MCE, whereas the SCR-U1.8-LR model correctly classified the patient as MCE. This case illustrates how the incorporation of CSF reserve may help identify high-risk patients even when lesion volume alone underestimates the overall severity of the infarction.

## 4. Discussion

This pilot study evaluated SCR-U1.8 as a single, physiologically interpretable imaging biomarker that integrates stroke lesion burden and cerebrospinal fluid reserve. Across 100 repeated stratified train–test splits, SCR-U1.8-LR achieved the highest accuracy, precision, specificity, F1 score, ROC-AUC, and average precision among the four evaluated prediction strategies. Compared with U1.8 alone, normalization of lesion volume by CSFV reduced false-positive classifications while maintaining high, although not perfect, sensitivity. These findings suggest that the balance between lesion burden and available CSF reserve may provide more informative exploratory risk stratification than lesion volume alone.

The results also demonstrate the limitations of relying exclusively on fixed infarct-volume thresholds. The U1.8 > 82 mL criterion achieved perfect sensitivity but had relatively low precision, indicating that many patients with large diffusion-abnormal lesions did not subsequently develop MCE. Increasing the threshold to >145 mL improved specificity and precision but reduced sensitivity, reflecting the expected trade-off between false-positive and false-negative classifications. The continuous U1.8-LR model showed intermediate performance but did not substantially resolve this limitation. In contrast, SCR-U1.8-LR achieved markedly higher precision and specificity than the volume-based strategies, suggesting that normalization by CSFV may help distinguish patients with similar lesion burdens but different capacities to accommodate subsequent swelling.

The physiological rationale for SCR-U1.8 is consistent with the Monro–Kellie framework [19]. U1.8 reflects the burden of diffusion-abnormal and potentially swollen brain tissue, whereas CSFV represents a component of the intracranial compensatory space available to accommodate subsequent swelling. SCR-U1.8 integrates these components into a single ratio, with higher values indicating greater lesion burden relative to available CSF reserve. Accordingly, patients with similar lesion volumes may differ in their capacity to tolerate edema depending on the amount of preserved CSFV.

The representative cases illustrate this concept. In Figure 3, a patient with a U1.8 lesion volume exceeding both conventional thresholds did not develop MCE. The U1.8 > 82 mL threshold, U1.8 > 145 mL threshold, and U1.8-LR model all produced false-positive classifications, whereas SCR-U1.8-LR correctly classified the patient as non-MCE because the relatively preserved CSFV reduced the lesion-to-CSF ratio. A complementary MCE case in Figure 4 may further illustrate how reduced CSF reserve and an elevated SCR-U1.8 can identify risk when lesion-volume-based strategies provide an equivocal or false-negative classification. Together, these examples support the clinical interpretability of SCR-U1.8 as a measure of lesion burden relative to compensatory reserve.

The comparison between SCR-U1.8-LR and the U1.8 > 82 mL threshold requires balanced interpretation. The fixed 82 mL threshold retained perfect sensitivity, whereas the mean sensitivity of SCR-U1.8-LR was 0.900. Therefore, SCR-U1.8-LR should not be interpreted as uniformly superior across all performance domains. Its principal advantage was the marked improvement in precision and specificity, with a corresponding reduction in false-positive classifications. In a clinical setting, this trade-off could potentially reduce unnecessary escalation of monitoring or intervention among patients with large infarcts who retain sufficient CSF reserve. However, the consequences of missing an MCE event are substantial, and the acceptable balance between sensitivity and specificity must be established prospectively.

The lower relative variation in the training-derived SCR-U1.8 cut point compared with the corresponding U1.8 cut point suggests less sensitivity to the specific composition of the training subset. However, because the repeated splits reused overlapping patients and each training subset included only eight MCE cases, this apparent partition-to-partition stability should be interpreted as exploratory and requires external validation.

Earlier studies, including those by Oppenheim et al. [9] and Thomalla et al. [10], used fixed infarct-volume thresholds to identify patients at risk for malignant cerebral edema. These methods were derived within specific hyperacute imaging windows and may not fully account for individual differences in intracranial compensatory reserve. In routine clinical practice, however, MRI may be performed beyond the immediate hyperacute phase, as reflected by the mean onset-to-MR interval of approximately 1.6–1.8 days in our cohort. Therefore, risk stratification based on lesion volume alone may be insufficient in patients imaged across broader time windows. The present study addressed this limitation by evaluating SCR-U1.8, which normalizes diffusion-abnormal lesion volume by CSFV and thereby incorporates both stroke lesion burden and available CSF reserve within a single imaging biomarker.

Table 5 summarizes representative imaging-based machine learning studies for MCE prediction. Previous studies have predominantly used CT-based imaging, manual lesion segmentation, and combinations of clinical, radiomic and treatment-related variables [2,20,21,22,23]. Direct numerical comparison should be interpreted cautiously because of differences in cohort composition, MCE prevalence, imaging modality, outcome definition, predictor selection, and validation strategy. In contrast, the present study used a fully automated DWI/ADC-based pipeline to quantify U1.8 lesion volume and CSFV without manual lesion delineation. The resulting SCR-U1.8 biomarker provides a simple, tissue- and reserve-aware representation of lesion burden relative to available CSF space. This low-complexity approach may facilitate integration into imaging workflows, although prospective multicenter validation remains necessary.

In clinical practice, initial DWI is routinely obtained early after hospital presentation [24], whereas follow-up CT is used to monitor edema progression and detect malignant cerebral edema [25]. The proposed framework is designed to operate at the time of initial MRI, providing early risk stratification before overt mass effect develops [26]. This timing is clinically relevant, as it precedes critical decision points such as escalation to neurocritical care monitoring, implementation of aggressive medical management, or consideration of decompressive hemicraniectomy [27,28,29]. By identifying patients at elevated risk before clinical or radiographic deterioration, the framework may support earlier clinical triage and more informed resource allocation [30]. Importantly, this imaging-based approach does not rely on treatment variables or clinical scores, allowing risk estimation to be generated directly from imaging data alone. This design may facilitate future integration into radiology workflows, pending external validation. However, the present study evaluated radiological MCE rather than patient-centered clinical outcomes, such as mortality or long-term functional disability. Although the early identification of patients at risk for MCE may facilitate closer monitoring, repeat imaging, neurocritical care triage, and timely neurosurgical consultation, the present study does not demonstrate that the proposed framework improves patient outcomes or changes treatment decisions. Future multicenter studies should evaluate whether implementation of this imaging-based framework for early risk stratification using reserve-aware imaging biomarkers translates into improved mortality, functional outcomes, and clinical decision-making.

Several limitations must be acknowledged. First, only 12 patients developed MCE. Although performance was evaluated across 100 repeated train–test splits, the same events recurred across repetitions, and each testing subset contained only four MCE cases. Consequently, a single misclassification could substantially affect sensitivity and other event-dependent metrics, and the performance estimates and candidate cut points may remain unstable or optimistic. Second, random undersampling reduced the number of non-MCE training candidates from 345 to 24 patients in each repetition. Although this improved class balance during model fitting, it discarded substantial majority-class information and may have increased model variability. Alternative strategies, including class weighting and penalized regression, should be evaluated in larger datasets. Third, SCR-U1.8 contains U1.8 in its numerator. Accordingly, the present findings do not establish that CSFV independently predicts MCE beyond lesion volume. Rather, they suggest that normalization of lesion burden by CSFV may provide a useful composite representation of lesion burden relative to reserve. Fourth, the study was retrospective and conducted at a single center. Baseline MRI and follow-up imaging were obtained according to clinical judgment rather than a predefined protocol, potentially introducing selection bias and limiting generalizability. Fifth, radiological MCE was used as the primary outcome. Although radiological swelling is clinically relevant, the present analysis does not demonstrate improvement in mortality, long-term functional outcome, quality of life, or treatment decision-making. Finally, the paired statistical comparisons across the 100 repetitions should be interpreted as exploratory because the repeated splits reused the same underlying patients and therefore were not statistically independent samples. The magnitude and consistency of paired performance differences are more informative than the Bonferroni-adjusted *p* values alone. Larger multicenter studies should prospectively validate the SCR-U1.8 candidate cut point, compare it with continuous U1.8 and established volume thresholds, and determine whether its use improves clinically meaningful patient outcomes.

## 5. Conclusions

In conclusion, SCR-U1.8 provides a simple and physiologically interpretable representation of stroke lesion burden relative to CSF reserve. Across repeated stratified train–test splits, SCR-U1.8-LR showed substantially higher precision and fewer false-positive classifications than U1.8-based strategies, although it did not preserve the perfect sensitivity achieved by the U1.8 > 82 mL threshold. These findings remain exploratory and require external validation before clinical implementation.

## Figures and Tables

**Table 1:** Demographics of Acute Ischemic Stroke Patients with Malignant Cerebral Edema (MCE) and non-MCE.

Characteristics	MCE	Non-MCE	*p* Value
Number (%)	12 (2.26%)	518 (97.74%)	
Female, *n* (%)	6 (50%)	192 (37.1%)	0.378
Age (years)	70 ± 18	68 ± 14	0.667
OMI (days)	1.75 ± 1.06	1.63 ± 1.17	0.657
Midline shift (*n*)	12	0	
Decompressive hemicraniectomy (*n*)	3	0	

Note: MCE denotes malignant cerebral edema, OMI denotes onset-to-MR interval, *n* denotes number, and age and OMI are presented as mean ± standard deviation. OMI did not differ significantly between MCE and non-MCE groups (1.75 ± 1.06 vs. 1.63 ± 1.17 days, *p* = 0.657), indicating comparable imaging timing between groups. All patients who underwent decompressive hemicraniectomy also fulfilled the radiological criterion of midline shift >5 mm.

**Table 2:** Comparison of Feature Characteristics between Malignant Cerebral Edema (MCE) and non-MCE Groups.

Characteristics	MCE (*n* = 12)	Non-MCE (*n* = 518)	*p* Value
U1.8 (mL)	284.48 ± 147.47	15.75 ± 36.57	<0.001
CSFV (mL)	82.99 ± 50.46	198.96 ± 73.28	<0.001
SCR-U1.8	5.26 ± 5.40	0.10 ± 0.26	<0.001

Note: U1.8 denotes U-Net-segmented stroke lesion volume defined using an ADC threshold of <1.8 × 10^−3^ mm^2^/s. CSFV denotes cerebrospinal fluid volume; SCR-U1.8 = U1.8/CSFV. Data are presented as mean ± standard deviation. *p* values < 0.001 are reported as <0.001.

**Table 3:** Training-Derived Cut Points and Empirical Stability Intervals for U1.8- and SCR-U1.8-Based Prediction of Malignant Cerebral Edema.

Prediction Strategy	Cut-Point Source	Median Cut Point	Empirical Stability Interval	Median Training Youden J	Youden J Stability Interval
U1.8 > 82 mL	Prespecified literature threshold	82 mL	NA	NA	NA
U1.8 > 145 mL	Prespecified literature threshold	145 mL	NA	NA	NA
Continuous U1.8	Training-derived raw-feature Youden index	106.89 mL	[106.89–169.70 mL]	0.957	[0.942–0.986]
Continuous SCR-U1.8	Training-derived raw-feature Youden index	1.43	[1.43–1.49]	0.994	[0.988–0.997]

Note: U1.8 denotes stroke lesion volume defined using an apparent diffusion coefficient (ADC) threshold of <1.8 × 10^−3^ mm^2^/s. CSFV denotes cerebrospinal fluid volume, and SCR-U1.8 denotes the ratio of U1.8 lesion volume to CSFV (U1.8/CSFV). The 82 mL and 145 mL thresholds were prespecified literature-based fixed cut points. The reported cut points refer to thresholds on the original continuous features rather than logistic-regression predicted probabilities. For the continuous U1.8 and SCR-U1.8 predictors, raw-feature cut points were selected exclusively within the training subset of each of the 100 repeated stratified train–test splits by maximizing the Youden index and were then applied unchanged to the corresponding testing subset. Cut points and training Youden J values are presented as medians with empirical 2.5th–97.5th percentile stability intervals in brackets. Because the repeated splits reused overlapping patients, these intervals quantify variation across data partitions and should not be interpreted as confidence intervals derived from independent cohorts. NA, not applicable.

**Table 4:** Performance Comparison of Four Prediction Strategies Across 100 Repeated Stratified Train–Test Splits.

Metric	U1.8 > 82 mL	U1.8 > 145 mL	Training-Derived U1.8 Threshold	Training-Derived SCR-U1.8 Threshold
ACC	0.946 ± 0.013 ***	0.972 ± 0.009 ***	0.956 ± 0.013 ***	0.990 ± 0.006
PRE	0.304 ± 0.058 ***	0.448 ± 0.108 ***	0.339 ± 0.084 ***	0.759 ± 0.150
SEN	1.000 ± 0.000 ***	0.810 ± 0.151 **	0.887 ± 0.196	0.900 ± 0.181
SPE	0.945 ± 0.013 ***	0.975 ± 0.009 ***	0.958 ± 0.015 ***	0.992 ± 0.005
F1	0.463 ± 0.065 ***	0.569 ± 0.108 ***	0.478 ± 0.095 ***	0.806 ± 0.131
AUC	NA	NA	0.988 ± 0.007 ***	0.998 ± 0.002
NPV	1.000 ± 0.000 ***	0.996 ± 0.004 ***	0.997 ± 0.005	0.998 ± 0.004
AP	NA	NA	0.749 ± 0.119 ***	0.935 ± 0.062

Note: Values are presented as mean ± standard deviation across 100 repeated stratified train–test splits. U1.8 denotes stroke lesion volume defined using an apparent diffusion coefficient (ADC) threshold of <1.8 × 10^−3^ mm^2^/s. CSFV denotes cerebrospinal fluid volume, defined using ADC values > 1.8 × 10^−3^ mm^2^/s within the intracranial compartment. SCR-U1.8 was calculated as U1.8/CSFV. U1.8-LR and SCR-U1.8-LR denote single-predictor logistic regression models using continuous U1.8 and SCR-U1.8, respectively. Asterisks in the U1.8 > 82 mL, U1.8 > 145 mL, and U1.8-LR columns indicate paired comparisons with SCR-U1.8-LR: ** Bonferroni-adjusted *p* < 0.01; *** Bonferroni-adjusted *p* < 0.001. The absence of an asterisk indicates a Bonferroni-adjusted *p* value ≥ 0.05. Adjustment was performed across all six pairwise comparisons among the four prediction strategies for each performance metric. Only comparisons with SCR-U1.8-LR are displayed in the table. Because the repeated splits reused the same underlying patients, the adjusted *p* values should be interpreted as exploratory. ACC, accuracy; PRE, precision; SEN, sensitivity; SPE, specificity; F1, F1 score; AUC, area under the receiver operating characteristic curve; NPV, negative predictive value; AP, average precision, NA, not applicable.

**Table 5:** Summary of Representative Imaging-Based Machine Learning Models for Prediction of Malignant Cerebral Edema.

Author	Cohort Size			Imaging Modality	Segmentation	Input Features	ML Model	Reported Performance						
	Total	MCE	Non-MCE					AUC	ACC	SEN	SPE	PRE	NPV	F1
Foroushani [2]	598	20	578	CT	Auto ^†^	Clinical + Imaging	FCN	0.980	0.930	0.950	0.930	0.330	NA	0.490
					Auto ^†^	Clinical + Imaging	LSTM	0.998	0.990	1.000	0.990	0.870	NA	0.930
					Auto ^†^ + Manual ^‡^	Clinical + Imaging + midline shift + lesion volume	LSTM	0.998	0.990	0.950	0.990	0.860	NA	0.900
Zhang [22]	219	67	152	NCCT	Manual ^‡^	Clinical + Radiomics	MLP	0.872	0.842	0.885	0.750	0.865	0.750	NA
Wen [20]	111	32	79	NCCT + CTP	Manual ^‡^	Radiomics + ASPECTS + Ischemic core vol + DT > 3 s + IV-tPA	NA	0.879	NA	0.800	0.917	NA	NA	NA
Zhang [21]	179	48	131	NCCT	Manual ^‡^	Clinical + Radiomics	LR	0.916	0.861	0.750	0.875	NA	NA	NA
Zeng [23]	110	24	86	NCCT	Manual ^‡^	Clinical + Imaging + Treatment	LR-stacking	0.885	0.909	0.900	0.913	NA	NA	0.895
Present study	530	12	518	DWI + ADC	Auto ^§^	U1.8 and CSFV-derived SCR-U1.8	Single predictor LR	0.998	0.990	0.900	0.992	0.759	0.998	0.806

Note: MCE, malignant cerebral edema; AUC, area under the receiver operating characteristic curve; ACC, accuracy; SEN, sensitivity; SPE, specificity; F1, F1 score; NPV, negative predictive value; NCCT, non-contrast-enhanced computed tomography; CTP, CT perfusion; IV-tPA, intravenous tissue plasminogen activator; DT > 3 s, delay time > 3 s; MLP, multilayer perceptron; FCN, fully convolutional network; LSTM, long short-term memory; LR, logistic regression; LR-stacking, logistic regression–based stacking ensemble; NA, not available. Performance metrics are reported as presented in the respective studies and may refer to testing sets or independent validation cohorts where available. Direct numerical comparisons should be interpreted cautiously because of differences in cohort size, MCE prevalence, imaging modality, outcome definition, model inputs, and validation strategies. ^†^ Automatic segmentation of cerebrospinal fluid. ^‡^ Manual segmentation of stroke lesions by expert readers. ^§^ Fully automated quantification of U1.8 stroke lesion volume and CSFV.

## Data Availability

The original contributions presented in this study are included in the article/Supplementary material. Further inquiries can be directed to the corresponding author.

## Supplementary Materials

The following supporting information can be downloaded at https://www.mdpi.com/article/10.3390/diagnostics16152483/s1. Table S1: Definitions of Imaging and Clinical Data Elements Corresponding to National Institute of Neurological Disorders and Stroke Common Data Elements.

## Abbreviations

The following abbreviations are used in this manuscript:

ADC	apparent diffusion coefficient
AIS	acute ischemic stroke
CSFV	cerebrospinal fluid volume
DWI	diffusion-weighted imaging
MCE	malignant cerebral edema
LR	logistic regression
U1.8	stroke lesion volume defined using an ADC threshold of <1.8 × 10^−3^ mm^2^/s
SCR-U1.8	stroke-to-CSF ratio calculated as U1.8/CSFV
