# Supplementary material for: Automated Diffusion-Weighted MRI Analysis for Exploratory Risk Stratification of Malignant Cerebral Edema After Acute Ischemic Stroke
Source: Diagnostics (Basel). 2026 Aug 6;16(15):2483. doi: 10.3390/diagnostics16152483 (PMC13465561; doi:10.3390/diagnostics16152483)
Supplement: Supplementary file 1 [file diagnostics-16-02483-s001.zip › diagnostics-4327015-supplementary.pdf]

Supplementary Materials

Table S1. Definitions of Imaging and Clinical Data Elements Corresponding to National Institute of Neurological Disorders and Stroke Common Data Elements

Study Variable	NINDS CDE Variable Name	NINDS CDE Definition	Unit / Format	Notes / Modifications
Sex	Sex	Biological sex of participant (Male / Female)	Categorical	Matches CDE categories
Age	Age at stroke onset	Age of participant at time of stroke onset	Years	Matches CDE definition
Onset-to-MR interval	Time to Imaging	Time between symptom onset and MRI	Days	Direct measurement in days from clinical records
MCE status	Malignant Cerebral Edema Indicator	Presence of malignant cerebral edema based on predefined imaging and clinical criteria	Binary (Yes/No)	Composite measure—requires imaging + clinical confirmation
U1.8 (ADC < 1.8 volume)	DWI lesion volume (high ADC threshold)	Infarct lesion volume segmented at ADC < 1.8×10^{-3} mm²/s	mL	Derived variable—custom segmentation
CSFV	Cerebrospinal Fluid Volume	Total cerebrospinal fluid volume	mL	Derived via morphological processing
SCR-U1.8	U1.8 / CSFV ratio	Ratio of lesion volume (ADC < 1.8×10^{-3} mm²/s) to cerebrospinal fluid volume	Unitless	Not in CDE—custom engineered metric
Model type (LR)	Model architecture	Type of machine learning classifier used	Categorical	Not in CDE—algorithm metadata

Note: NINDS denotes National Institute of Neurological Disorders and Stroke, CDE denotes Common Data Elements.
